# Suicidal risk among adolescent psychiatric inpatients: the role of insomnia, depression, and social-personal factors

**DOI:** 10.1192/j.eurpsy.2025.29

**Published:** 2025-02-28

**Authors:** Valentina Baldini, Martina Gnazzo, Martina Maragno, Rebecca Biagetti, Camilla Stefanini, Francesco Canulli, Giorgia Varallo, Cristina Donati, Giovanni Neri, Andrea Fiorillo, Giuseppe Plazzi

**Affiliations:** 1 Department of Biomedical and Neuromotor Sciences, University of Bologna, Bologna, Italy; 2 Department of Biomedical, Metabolic and Neural Sciences, University of Modena and Reggio Emilia, Modena, Italy; 3 Private Accredited Hospital Villa Igea, Modena, Italy; 4 Department of Psychiatry, University of Campania “Luigi Vanvitelli”, Naples, Italy; 5 IRCCS Istituto Delle Scienze Neurologiche di Bologna, Bologna, Italy

**Keywords:** adolescent, depression, insomnia, sleep disturbances, suicide

## Abstract

**Background:**

Adolescents with psychiatric disorders are at increased risk of suicide, with insomnia, depression, and social-personal factors playing pivotal roles. This study investigates the interplay between these factors in a sample of adolescent psychiatric inpatients in Italy, with a particular focus on their association with suicide attempts.

**Methods:**

We conducted a cross-sectional study on 95 adolescent inpatients (54 suicide attempters, 41 non-attempters) to explore their sociodemographic and clinical variables, including insomnia, depression, and social-personal factors as history of bullying. Logistic regression analyses and Pearson’s correlations were used to identify significant predictors of suicide attempts and their interrelations.

**Results:**

Suicide attempters were predominantly female (90% vs. 75%, *p* = 0.04) and more likely to have a family psychiatric history (83% vs. 63%, *p* = 0.04), a history of bullying (26% vs. 9%, *p* = 0.01), and insomnia (79% vs. 53%, *p* = 0.01). Depression was strongly associated with suicide attempts (96% vs. 70%, *p* = 0.01), while physically active adolescents were significantly less likely to attempt suicide (27% vs. 53%, *p* = 0.01). Insomnia and depression were highly correlated (*r* = 0.94, *p* = 0.02), emphasizing the critical role of the former in emotional dysregulation. Behavioral factors, such as physical inactivity and bullying, emerged as additional key contributors to suicidal behavior.

**Conclusion:**

This study highlights the multifaceted nature of suicide risk in adolescent psychiatric inpatients, with sleep disturbances, depression, and behavioral factors playing central roles. These findings underscore the need for integrated interventions targeting sleep, emotional regulation, and behavioral vulnerabilities to mitigate suicide risk.

## Introduction

Adolescence is a critical transitional period characterized by significant physical, emotional, and social changes. This stage of development is also marked by heightened vulnerability to mental disorders, with nearly 20% of adolescents experiencing a diagnosable psychiatric condition each year [1, 2]. Suicide, now the second leading cause of death among adolescents globally, represents a growing public health crisis requiring urgent attention [3, 4]. A recent study shows that most adolescents who die by suicide have had contact with a healthcare provider in the year (88%) and even the month (42%) before their death, highlighting critical missed opportunities for early intervention [5]. Among the factors influencing adolescent mental health, insomnia has emerged as a critical area of focus due to its profound effects on emotional regulation, cognitive functioning, and overall psychological well-being [6]. Insomnia, characterized by persistent difficulties in initiating or maintaining sleep or early morning awakenings, is not only highly prevalent in adolescents but also strongly associated with increased risks of mood disorders and suicidal behavior [7–10]. Studies consistently show that insomnia contributes to emotional dysregulation by intensifying negative affect and diminishing the capacity to manage stress, both of which are key factors underlying suicidal ideation and behavior [11]. Moreover, insomnia often co-occurs with depressive symptoms, creating a bidirectional relationship where sleep disturbances exacerbate mood dysregulation, while depression further impairs sleep patterns. This interplay may amplify feelings of hopelessness and helplessness, which are recognized precursors of suicide risk in vulnerable adolescents [12]. These issues are significant because poor sleep is a modifiable, non-stigmatizing, and highly treatable factor, highlighting its potential as a target for intervention.

Depression, one of the most prevalent psychiatric disorders in adolescence, has long been recognized as a key contributor to suicidal behavior [13]. Depressed adolescents often exhibit feelings of worthlessness, hopelessness, and social withdrawal, which can act as precursors of suicidal ideation and attempts [14]. Depression is also strongly associated with other vulnerabilities, including non-suicidal self-injury (NSSI), substance use, and sleep disturbances, further complicating the clinical picture [15]. Given the complex interplay between these variables, there is a critical need for studies that simultaneously examine their combined influence on suicidality.

Behavioral and environmental factors, such as bullying, physical inactivity, and family dynamics, also play a pivotal role in adolescent suicidality. Bullying, both in-person and online, has been consistently associated with suicidal ideation and attempts, as it contributes to feelings of isolation, low self-esteem, and chronic stress [16, 17]. On the other hand, protective behaviors, such as regular physical activity, have been shown to improve mood, enhance emotional regulation, and reduce stress, offering a potential buffer against suicide risk [18].

Despite the growing body of evidence linking these factors to adolescent suicidality, much of the existing research has focused on general populations rather than high-risk groups, such as psychiatric inpatients. Adolescents hospitalized for psychiatric conditions represent a uniquely vulnerable population, as they often present with multiple co-occurring risk factors, including severe depression, chronic insomnia, and histories of trauma or abuse. Understanding the specific characteristics that distinguish suicide attempters from non-attempters in this high-risk group is essential for developing effective prevention and intervention strategies.

This study seeks to address these gaps by examining the interplay between insomnia, depression, and behavioral factors in a sample of adolescent psychiatric inpatients in Italy. Specifically, we aim to identify the sociodemographic, clinical, and behavioral characteristics associated with suicide attempts and explore how these factors interact to influence risk. By focusing on this high-risk population, our findings can provide valuable insights into the development of targeted, multifaceted interventions to reduce suicide rates among adolescents.

## Methods

### Study design and setting

This prospective study was conducted at the Children and Adolescents Intensive Treatment Ward, “Villa Igea—il Nespolo,” a tertiary-care facility specializing in intensive psychiatric treatment for adolescents. Participants were recruited between January 2020 and December 2024. Basic demographic information, including age and gender, was systematically recorded for all enrolled individuals.

Ethical approval was obtained from the Institutional Review Board of the Medical University of Modena and Reggio Emilia, Italy, and all procedures complied with relevant guidelines for human research. The study adhered to the Declaration of Helsinki guidelines for ethical research.

### Participants

Adolescents aged 12–18 years admitted for acute psychiatric care due to suicide attempts were eligible for inclusion. A suicide attempt is defined as a self-directed harmful behavior to end one’s life, regardless of the outcome, in line with DSM-5 criteria. In contrast, suicidal ideation is defined as recurrent or persistent thoughts of death or wishing to die without necessarily having a specific plan or intent to act on these thoughts. Patients were excluded if they had incomplete medical records, primary diagnoses of neurological disorders, or conditions directly affecting sleep independent of psychiatric symptoms (e.g., sleep apnea, narcolepsy).

### Data collection

Demographic and clinical data were extracted from electronic medical records. Psychiatric diagnoses, including depression and NSSI, were determined by attending psychiatrists using DSM-5 criteria during clinical interviews. Insomnia was defined as difficulty initiating or maintaining sleep or experiencing early morning awakenings, occurring at least three nights per week for a minimum of 1 month, accompanied by significant daytime impairment, according to the International Classification of Sleep Disorders, 3rd Edition criteria for insomnia.

Information about cannabis use and compulsive social media usage was gathered from electronic medical records, specifically documented during psychiatric intake interviews. Cannabis use was categorized as occasional or frequent based on patients’ self-reports and clinical assessments. Compulsive social media use was evaluated qualitatively by psychiatrists, who considered patients’ self-reported difficulties in managing their time on social media and its impact on daily functioning. During these clinical interviews, psychiatrists rated the presence of cannabis and social media misuse on a scale from 0 to 1.

Physical activity was classified as structured (e.g., participation in sports, gym workouts) or unstructured (e.g., walking, cycling), according to self-reported involvement in these activities.

These evaluations were part of the standard admission process and included detailed clinical interviews, patient history, and behavioral assessments.

Participants were divided into two groups: those with a reported suicide attempt (suicidal group) and those without (non-suicidal group). This classification was based on medical records and clinical evaluations conducted during hospitalization. Suicide risk was assessed clinically by attending psychiatrists based on structured clinical interviews and medical history as part of routine psychiatric evaluation. No specific psychometric scales were used for this assessment.

### Procedures

All clinical evaluations were performed during the first 48 hours of admission. Two independent researchers extracted information from electronic medical records to ensure data accuracy and cross-referencing findings to minimize errors. Any discrepancies were resolved through consensus discussions with a senior psychiatrist. A standardized protocol guided the data extraction process to maintain consistency, and regular quality checks were conducted to verify the completeness and reliability of the extracted variables.

### Statistical analysis

Descriptive statistics (means, standard deviations, and frequencies) were utilized to summarize participant characteristics. Chi-square tests or Fisher’s exact tests were employed to evaluate associations between insomnia and categorical variables, while independent *t*-tests or Mann–Whitney *U* tests were applied to continuous variables.

Spearman’s correlation coefficients were calculated to examine the relationships among insomnia, depression, NSSI, cannabis abuse, and compulsive social media use, considering the potential non-normal distribution of the data. Correlation coefficients (*r*) were reported with asterisks indicating statistically significant values, and the corresponding *p*-values (*p* < 0.05 or *p* < 0.01) were noted in the table legend for clarity.

A stepwise approach was employed for the logistic regression model to minimize multicollinearity, and independent variables were selected based on their clinical relevance and statistical significance from univariate analyses. Odds ratios (ORs) with 95% confidence intervals (CIs) were calculated to assess the strength of associations. Statistical significance was determined at *p* < 0.05. All analyses were carried out using JASP version 12.2.

## Results

### Socio-demographic and clinical characteristics

A total of 95 individuals were recruited, including 54 in the suicidal group and 41 in the non-suicidal group (Table 1). Female participants were more prevalent among suicide attempters (90%) than in no-suicide attempters (75%), showing a statistically significant difference (*p* = 0.04). The median age did not differ significantly between the groups, centered at 16 years (*p* = 0.40). Family income distributions were comparable across the groups, with no significant difference (*p* = 0.60). A higher proportion of suicide attempters had a family psychiatric history (83%) compared to no-suicide attempters (63%) (*p* = 0.04). In contrast, although more suicide attempters reported a family history of suicide (26%) compared to no-suicide attempters (9%), this difference did not reach statistical significance (*p* = 0.47).

**Table 1.** Sociodemographic and clinical characteristics of the sample

Variable	Suicide attempters (n = 54)	No-suicide attempters (n = 41)	*p* value	*χ*^2^
Female sex, n (%)	49 (90)	31 (75)	**0.04**	3.15
Age, median (IQR)	16 (1)	16 (3)	0.40	–
Family income, n (%)				2.26
Low	7 (12)	7 (17)	0.60	–
Lower-middle	37 (68)	22 (53)		–
Upper-middle	10 (18)	12 (29)		–
Family psychiatric history, n (%)	45 (83)	26 (63)	**0.04**	4.50
Family suicide history, n (%)	14 (26)	4 (9)	0.47	2.07
Bullied, n (%)	14 (26)	4 (9)	**0.01**	5.00
Physically active, n (%)	15 (27)	22 (53)	**0.01**	6.50
Tobacco use, n (%)	30 (55)	16 (39)	0.09	2.05
Cannabis use, n (%)	16 (29)	10 (24)	0.53	1.30
Alcohol use, n (%)	9 (16)	8 (19)	0.71	0.01
Depression, n (%)	52 (96)	29 (70)	**0.01**	7.50
NSSI, n (%)	49 (90)	34 (82)	0.15	1.60
Insomnia, n (%)	43 (79)	22 (53)	**0.01**	4.75
Initial insomnia	37 (68)	23 (56)	0.21	–
Maintenance insomnia	21 (38)	17 (41)	0.80	–
Terminal insomnia	5 (9)	5 (12)	0.64	–
Nighttime sleep duration, n (%)				0.45
<6 hours	33 (61)	27 (65)	0.68	–
6 hours	21 (38)	10 (24)		–
≥7 hours	0	4 (8)		–
Nightmares, n (%)	17 (31)	5 (12)	**0.02**	3.02

*p* < 0.05.

Insomnia was more common in suicide attempters (79%) than in non-suicide attempters (53%; *p* = 0.01). Although the subtypes of insomnia (initial, maintenance, and terminal) did not differ significantly between the groups, the frequency of nightmares was higher in suicide attempters (31%) compared to non-suicide attempters (12%; *p* = 0.02). Nighttime sleep duration showed no significant group differences (*p* = 0.68), with most participants in both groups reporting less than 6 hours of sleep per night.

Bullying was significantly more common among suicide attempters, with 26% reporting being bullied compared to 9% in the non-suicide attempters’ group (*p* = 0.01). Physical activity was less common in suicide attempters (27%) than in non-suicide attempters (53%; *p* = 0.01). Tobacco use was more prevalent among suicide attempters (55%) than in the other group (39%), although this difference was not statistically significant (*p* = 0.09). Similarly, cannabis and alcohol use did not differ significantly between the groups. Depression was reported by nearly all suicide attempters (96%) compared to 70% of non-suicide attempters, a statistically significant difference (*p* = 0.01).

### Correlation between insomnia and clinical variables

Spearman correlations were calculated to evaluate the relationship among insomnia, depression, NSSI, cannabis abuse, and compulsive social media use (Table 2). A strong positive correlation was found between insomnia and depression (*r* = 0.45, *p* < 0.05), suggesting that higher levels of insomnia correspond to increased depressive symptoms.

**Table 2.** Spearman correlation analysis between insomnia and clinical variables

Variable	Insomnia	Depression	NSSI	Cannabis abuse	Compulsive social media use
Insomnia	1.00	0.45*	0.10	−0.05	0.12
Depression	0.45*	1.00	0.40*	0.20	0.18
NSSI	0.10	0.40*	1.00	0.25*	0.22
Cannabis abuse	−0.05	0.20	0.25*	1.00	−0.10
Compulsive social media use	0.12	0.18	0.22*	−0.10	1.00

* *p* value < 0.05.

Depression exhibited a moderate positive correlation with NSSI (*r* = 0.40, *p* < 0.05), suggesting that increased depressive symptoms were linked to higher engagement in NSSI.

NSSI showed a weak to moderate positive correlation with cannabis abuse (*r* = 0.25, *p* < 0.05) and a moderate positive correlation with compulsive social media use (*r* = 0.22, *p* < 0.05), both indicating a statistically significant relationship.

These findings emphasize the significant connection between insomnia and depression, suggesting that NSSI may be related to both cannabis abuse and compulsive social media use. Conversely, other relationships appear weak or non-significant.

### Logistic regression

The logistic regression analysis identified several significant predictors of the outcome, as given in Table 3. Age was significantly associated with the outcome (*p* = 0.05), with each additional year increasing the odds by 11% (OR = 1.11).

**Table 3.** Logistic regression analysis

Variable	Coefficient (β)	Standard error	*p* value	Odds ratio
Age	0.10	0.05	**0.05**	1.11
Gender	0.22	0.15	0.12	1.24
Family psychiatric history	0.33	0.19	0.07	1.39
Insomnia	0.55	0.20	**0.01**	1.73
Economic status	−0.10	0.08	0.18	0.90
Physically active	−0.42	0.18	**0.02**	0.66
Depression	0.85	0.28	**0.01**	2.34

*p* < 0.05.

Insomnia emerged as a significant predictor (*p* = 0.01), with individuals experiencing insomnia having 73% higher odds of the outcome (OR = 1.73). Similarly, depression was strongly associated with the outcome (*p* = 0.01), with individuals diagnosed with depression being more than twice as likely to experience the outcome (OR = 2.34).

Physical activity demonstrated a protective effect (*p* = 0.02), reducing the odds of the outcome by 34% (OR = 0.66). Family psychiatric history showed a trend toward significance (*p* = 0.07), with individuals having a family history of psychiatric conditions being 39% more likely to experience the outcome (OR = 1.39).

Conversely, gender (*p* = 0.12, OR = 1.24) and economic status (*p* = 0.18, OR = 0.90) were not statistically significant predictors in this model.

These findings highlight the significant roles of age, insomnia, depression, and physical activity in influencing the outcome, with physical activity serving as a protective factor. While family psychiatric history showed a potential association, gender and economic status were not significant predictors.

## Discussion

This study highlights the complex relationships between insomnia, depression, physical activity, and familial factors in adolescent psychiatric inpatients. The findings underscore the interplay of behavioral and psychological variables in shaping mental health outcomes, with significant clinical implications.

Female adolescents accounted for a significantly higher proportion of suicide attempters compared to non-attempters. This finding aligns with prior research demonstrating that females are more likely to attempt suicide, while males tend to complete suicide at higher rates [19]. Possible explanations include greater susceptibility to internalizing disorders, such as depression and anxiety, as well as higher exposure to social stressors, including bullying and interpersonal conflicts [20]. These findings suggest the need for gender-specific interventions that address the unique risk factors faced by female adolescents.

Insomnia has been identified as a significant predictor of adverse psychiatric outcomes, with adolescents experiencing insomnia demonstrating more than double the likelihood of such challenges. This sleep disorder has a well-documented bidirectional relationship with mental health conditions, intensifying symptoms of anxiety, depression, and emotional dysregulation [21–23]. Physiologically, insomnia disrupts the normal functioning of the hypothalamic-pituitary-adrenal axis, resulting in heightened stress reactivity and impaired emotional regulation [24].

In adolescent psychiatric inpatients, addressing insomnia is particularly critical, as it may substantially influence the effectiveness of treatment interventions [25]. Implementing targeted strategies to improve sleep, such as cognitive-behavioral therapy for insomnia (CBT-I), has shown promise in reducing the impact of sleep disturbances and enhancing overall therapeutic outcomes. Incorporating CBT-I into routine care for adolescents could play a pivotal role in mitigating the negative effects of insomnia on mental health and promoting recovery [26].

Depression was the strongest predictor of adverse outcomes, with adolescents diagnosed with depression being more than three times as likely to experience poor outcomes. This aligns with prior research demonstrating the pervasive effects of depression on cognitive, emotional, and behavioral functioning [27]. In adolescents, depression is often accompanied by heightened feelings of hopelessness, reduced motivation, and difficulties in engaging in treatment, which can complicate recovery [28]. Early identification and aggressive management of depressive symptoms are critical. Multimodal approaches combining pharmacological treatments, evidence-based psychotherapies, and family involvement have shown promise in reducing symptom severity and improving long-term outcomes [29].

Several behavioral factors emerged as significant correlates of suicide attempts. Notably, bullied adolescents were far more likely to be suicide attempters. Bullying has been consistently linked to suicidal ideation and attempts in adolescents, as it contributes to feelings of isolation, helplessness, and low self-worth [30]. This finding reinforces the importance of school-based anti-bullying programs and early detection of victimization to prevent suicidal behavior.

Physical activity was found to be significantly lower in suicide attempters, further supporting the protective role of exercise in mental health. Physical activity is believed to exert these effects through multiple mechanisms, including the release of endorphins, modulation of stress hormones, and anti-inflammatory effects [31]. In inpatient settings, incorporating structured physical activity programs may provide a low-cost, non-pharmacological adjunct to traditional psychiatric care. Furthermore, such programs could be tailored to individual preferences to ensure adherence and maximize psychological benefits.

Adolescents with a family history of psychiatric conditions were 49% more likely to experience adverse outcomes. This finding highlights the importance of genetic and environmental influences in shaping mental health trajectories. Previous studies have shown that familial psychiatric history contributes to the heritability of mental disorders and can also impact adolescents through learned maladaptive coping mechanisms or increased exposure to family stressors [32]. Given this, clinicians should prioritize collecting comprehensive family histories during intake assessments. This information can inform risk stratification and the development of personalized treatment plans that account for potential genetic and environmental vulnerabilities.

In this study, neither gender nor economic status was significantly associated with psychiatric outcomes. While prior research suggests that these factors can influence mental health outcomes, particularly in community-based samples, their impact may be attenuated in inpatient populations due to the severity of underlying psychiatric illnesses [33]. Gender differences, for instance, may be less pronounced in settings where both male and female adolescents experience comparable levels of distress. Similarly, the uniformity of socioeconomic factors within this Italian sample may have limited variability, reducing the detectability of any significant associations.

The moderate correlation between NSSI and compulsive social media use supports the growing evidence that excessive social media use may contribute to maladaptive coping mechanisms in vulnerable adolescents [34, 35]. Social media platforms often expose users to content that can normalize or glorify self-injurious behaviors, reinforcing harmful practices [36]. Moreover, excessive engagement in social media can contribute to sleep disruptions, which, in turn, may exacerbate underlying mental health conditions [37]. Interventions promoting healthy digital habits and fostering media literacy could mitigate this risk.

Interestingly, the negative correlation between cannabis abuse and compulsive social media use was not statistically significant, suggesting that these behaviors may represent distinct coping strategies for managing psychological distress. Cannabis abuse has often been associated with social withdrawal and avoidance of interpersonal connections, whereas compulsive social media use may reflect a heightened need for social validation and connection [38, 39]. Future studies could investigate whether these patterns are influenced by underlying personality traits, such as introversion or extraversion, to understand their clinical implications better.

The weak and non-significant association between insomnia and NSSI contrasts with prior studies suggesting stronger links between sleep disturbances and self-injurious behaviors [40]. One possible explanation is the mediating role of emotional dysregulation or interpersonal stress, which may vary across clinical samples. Furthermore, different forms of NSSI (e.g., cutting vs. burning) could exhibit distinct relationships with sleep patterns, warranting further exploration [41, 42].

This study has several limitations that should be considered when interpreting the findings. First, the cross-sectional design precludes establishing causal relationships between the identified factors and suicidal behavior. Longitudinal studies are needed to confirm the temporal dynamics and causal pathways among sleep disturbances, depression, and suicidal behavior.

Second, psychometrically validated instruments are not used to assess the intensity or severity of the symptoms. This poses a significant limitation, as the lack of standardized tools may impair the objectivity and accuracy of the findings. Using established psychometric scales would have produced a more precise and dependable assessment.

Third, the sample size, while sufficient for detecting significant associations, limits the generalizability of the findings, particularly for non-significant results and subgroup analyses. Additionally, the sample was drawn from a single tertiary care center in Italy, which may not fully reflect the characteristics of adolescent psychiatric populations in other geographic or cultural contexts.

Finally, the study did not account for potential mediating or moderating factors, such as the severity of comorbid psychiatric conditions, trauma history, or the duration and treatment of sleep disturbances. Including these variables in future research could provide a more nuanced understanding of the interplay between behavioral, psychological, and environmental factors in adolescent suicidality.

However, our findings reinforce the importance of understanding the broader psychosocial context when addressing mental health problems in adolescents. Sleep disturbances, substance use, and digital behaviors should not be considered in isolation but rather as interrelated factors that collectively influence psychological well-being.

## Conclusion

The significant correlations identified in this study highlight key areas for clinical intervention and further research. Addressing insomnia and depressive symptoms simultaneously, promoting healthy social media use, and understanding individual coping strategies are essential steps in improving mental health outcomes in adolescents. Interdisciplinary collaboration, integrating psychiatry, psychology, and sleep medicine specialists, will be critical in advancing care for this vulnerable population.

## Data Availability

Data are available upon request from the corresponding author.
